# Phytic acid and grape seed extract in dentin biomodification: effects on bond strength and interface integrity

**DOI:** 10.3389/fdmed.2026.1921978

**Published:** 2026-08-26

**Authors:** Astrid Ana Gomes, Darshana Devadiga, Roma M, Darshan Madihalli Chandramouli, Rahul D. Rao, Manikandan Ravinanthanan, Dheeraj Devadiga

**Affiliations:** 1 Department of Conservative Dentistry and Endodontics, Nitte (Deemed to be University) AB Shetty Memorial Institute of Dental Sciences (ABSMIDS), Mangalore, Karnataka, India; 2 Department of Conservative Dentistry and Endodontics, Manipal College of Dental Sciences Mangalore, Manipal Academy of Higher Education, Manipal, India; 3 Department of Orthodontics and Dentofacial Orthopaedics, Nitte (Deemed to be University) AB Shetty Memorial Institute of Dental Sciences (ABSMIDS), Mangalore, Karnataka, India; 4 Dept of Conservative Dentistry and Endodontics, Bharati Vidyapeeth Deemed to be University Dental College & Hospital, Mumbai, Maharashtra, India; 5 Faculty of Dentistry, Restorative Dental Sciences, Al Baha University, Al Baha, Saudi Arabia; 6 Dept of Oral & Maxillofacial Surgery, Century International Institute of Dental Sciences and Research Center, Kasargod, Kerala, India

**Keywords:** cross-linkers, dentine, grape seed extract, hybrid layer, phytic acid, shear bond strength

## Abstract

**Background:**

Hybrid layer degradation, driven by residual matrix metalloproteinase (MMP) activity and hydrolytic attack at the resin–dentin interface, remains a primary cause of adhesive restoration failure. Dentin biomodification using natural cross-linking agents represents a promising strategy to enhance collagen stability and inhibit enzymatic degradation of the bonded interface.

**Objective:**

To evaluate and compare the effect of collagen cross linking agents, phytic acid (IP6) and grape seed extract (GSE), to reinforce the shear bond strength (SBS) of resin composite to dentin.

**Materials and methods:**

Enamel of 34 human maxillary premolars was eliminated and each tooth was sectioned to make a total sample size of 68 which was divided into four groups (*n* = 17) for surface treatment: G1-PA:37% Phosphoric acid (control), G2-IP6:1% phytic acid, G3-PA + GSE:37% phosphoric acid+6.5% GSE and G4-IP6 + GSE:1% phytic acid+6.5% GSE. A hybrid resin composite material was bonded and subjected to 1,000 cycles of thermocycling followed by SBS testing and analysis of fracture mode by Scanning Electron Microscopy. The data was statistically analysed using one-way Analysis of Variance (ANOVA) followed by Tukey's Honestly Significant Difference (HSD) *post hoc* test.

**Results:**

G4-IP6 + GSE showed the highest SBS (23.44 ± 4.28 MPa), followed by G2-IP6 (21.51 ± 6.00 MPa) and G3-PA + GSE (21.01 ± 4.35 MPa), while G1-PA had the lowest values of 15.40 ± 4.60 MPa. FTIR analysis showed that phytic acid exhibited a broad peak at 3311.77 cm⁻^1^ while GSE showed peaks 3266.37 cm⁻^1^ and 1602.93 cm⁻^1^ corresponding to hydroxyl and carbonyl functional groups.

**Conclusion:**

Phytic acid and grape seed extract significantly improved the composite–dentin shear bond strength of dentin. Their application appears to enhance the structural integrity of demineralized collagen at the resin–dentin interface, thereby supporting their potential incorporation into adhesive restorative protocols as a promising strategy to enhance dentin bond strength.

## Introduction

Adhesive bonding to enamel and dentin primarily relies on the modification of the dental substrate to facilitate the infiltration and subsequent polymerization of resin monomers. This has traditionally been accomplished using acid etchants or self-etch adhesive systems containing acidic functional monomers, which partially demineralize the substrate and facilitate resin infiltration and micromechanical interlocking. Enamel, owing to its mineral-rich composition, responds favourably to phosphoric acid (PA) etching, which has long been considered the gold standard for enamel bonding (1). In contrast, dentin is a more complex and heterogeneous substrate, containing a greater proportion of organic matrix and water, and therefore requires a more conservative etching approach comprising of shorter etching durations and milder acidic conditioners.

The unique structural and compositional characteristics of dentin present several challenges to the formation of a durable resin–dentin bond. Following acid conditioning, dissolution of the smear layer and smear plugs increases dentinal tubule patency and fluid movement, thereby complicating moisture control at the bonding interface. Excessive moisture may dilute adhesive components and interfere with resin infiltration, whereas excessive drying can induce collapse of the exposed collagen network, restricting monomer penetration and compromising the formation of a well-infiltrated hybrid layer (2, 3). Furthermore, dentinal tubules establish a direct anatomical communication between the external dentin surface and the pulp, and their exposure during adhesive procedures may contribute to fluid movement and postoperative sensitivity. These characteristics highlight the importance of establishing a stable and well-sealed resin–dentin interface (2, 4, 5).

Despite the initial formation of an apparently intact resin–dentin interface, its long-term stability remains inherently susceptible to degradation. Incomplete infiltration of resin monomers into the acid-demineralized dentin matrix can leave collagen fibrils insufficiently encapsulated and exposed to degradation. PA etching of dentin also has the potential for activation of endogenous proteolytic enzymes such as matrix metalloproteinases (MMPs), produced by odontoblasts that remain trapped within the calcified collagen matrix, triggering the enzymatic breakdown of interfacial collagen, deteriorating the resin-dentin bond (5, 6). In addition to these endogenous degradation mechanisms, the hybrid layer at the dentin–restorative interface is exposed to external challenges, including thermal fluctuations and masticatory stresses, which may contribute to interfacial deterioration and facilitate chemical or microbial micro leakage (2, 7). Thus, the degradation of the resin–dentin interface is not attributable to a single mechanism but represents the cumulative consequence of incomplete resin infiltration, collagen degradation, enzymatic activity, and long-term hydrolytic and mechanical challenges.

Dentin biomodification is a modern biomimetic strategy proposed by Bedran-Russo et al. (8) to mechanically strengthen the existing collagen network by altering its biochemistry and biomechanical properties using collagen cross-linking agents and modulation of endogenous matrix-degrading enzymes in an attempt to simultaneously improve the structural stability and biodegradation resistance of the dentin organic matrix (8).

Natural collagen cross-linking agents, such as proanthocyanidins and genipin, along with synthetic agents like glutaraldehyde and carbodiimide, have been investigated for this purpose (8). Among these, Grape seed extract (GSE), is a well-established and widely studied collagen cross-linker, rich in proanthocyanidins, and reported to inhibit more than 90% MMPs, leading to the formation of a more rigid collagen matrix that resists degradation over time (9, 10). However, the primary function of GSE is collagen biomodification rather than dentin conditioning, highlighting the potential advantage of a multifunctional agent capable of simultaneously conditioning the dentin substrate and biomodifying the underlying collagen matrix, thereby providing an integrated approach within a single treatment.

Phytic acid (IP6), a naturally occurring inositol derivative abundant in plant seeds, displays a strong negative charge density, imparting a chelating effect on multivalent cations like calcium to expose the underlying collagen network in preparation to receive resin monomers, thereby raising its potential for evaluation as a dentin conditioner (11). In addition to this, it has demonstrated interactions with specific proteins, resulting in stabilized cross-linked protein nanofibers and inhibitory effects on endogenous enzymatic activity, suggesting that its role may extend beyond conventional dentin conditioning (11).

While the individual effects of IP6 and GSE on dentin bond strength have been studied independently, their complementary characteristics provide a biologically plausible rationale for their sequential application, representing a novel approach. IP6 may facilitate conditioning of the dentin substrate and expose the collagen matrix, while the subsequent application of GSE may promote further collagen cross-linking and enhance resistance to enzymatic degradation. Such a conditioning–biomodification strategy could theoretically address two interconnected determinants of resin–dentin bond durability: effective modification of the dentin substrate to facilitate resin infiltration and the preservation of the collagen framework supporting the hybrid layer.

Therefore, this experimental study aimed to evaluate the effects of IP6 and GSE, separately and sequentially, on the shear bond strength (SBS) of resin composite to dentin and failure mode analysis of the de-bonded surfaces by Scanning Electron Microscopy (SEM) imaging. Fourier Transform Infrared spectroscopy (FTIR) of IP6 and GSE compounds was done to characterize chemical composition profile to elucidate their mechanism of interaction with dentin collagen.

The null hypothesis was that dentin pretreatment with IP6 and GSE would not significantly affect the shear bond strength compared with conventional PA conditioning.

## Materials and methods

### Preparation of samples

34 freshly extracted human maxillary premolars, extracted for orthodontic purposes, were collected, screened and disinfected. Exclusion criteria: Teeth with visible cracks on transillumination; teeth with dentinal hypersensitivity history; teeth exhibiting enamel fluorosis grade ≥2 (Dean's index); teeth from patients with systemic conditions known to affect dentin composition.

The outer enamel from all teeth was eliminated using a diamond disc and dentin from buccal/lingual surface was ground flat using 180, 320 and 600 grades of silicon carbide papers to simulate the surface characteristics produced by clinical diamond or carbide burs, resulting in a standardized, reproducible smear layer of 1.5 ± 0.5 µm thickness across all specimens. Finally, each tooth was mesio-distally sectioned to obtain two specimens each to obtain a total of 68 samples which were randomly allocated into the experimental groups (11). Sample size was determined *a priori* for four experimental groups, assuming a large effect size (f = 0.8), an alpha level of 0.05, and 80% statistical power, yielding a minimum requirement of 17 specimens per group. This study was approved by the Institutional Ethical Committee (approval no. ETHICS/ABSMIDS/514/2024).

### Preparation of collagen stabilizing solutions

1% IP6 solution was freshly prepared by diluting Phytic Acid solution (Tokyo Chemical Industry, Tokyo, Japan) in distilled water to achieve a pH of 1.2 that was verified using a digital pH meter.

6.5% GSE solution was prepared by mixing 6.5 g of ground GSE powder derived from a commercial grape seed extract supplement (HealthyHey Nutrition; HealthyHey Foods LLP., Maharashtra, India) with 100 mL of distilled water to make a pH of 4 (approximately).

### Dentin pretreatment and bonding

The total sample size (*n* = 68) was randomly distributed into four groups (*n* = 17) for dentin surface treatments: *G1-PA* (Control): PA 37% for 15 s (Septodont, Saint-Maur-des-Fossés, France), G2-*IP6:* IP6 1% for 30 s, *G3-PA* *+* *GSE:* PA 37% + GSE 6.5% (PA for 15 s followed by GSE for 1 h) and *G4-IP6* *+* *GSE:* IP6 1% + GSE 6.5% (IP6 for 30 s followed by GSE for 1 h). All treated samples were rinsed with distilled water for 30 s and an ethanol-water-based bonding agent were applied on the dentin surface (Adper Single Bond 2, 3M ESPE) in two coats that were each gently air-dried for 5 s and light-cured for 10 s at 1000 mW/cm^2^ (Woodpecker iLED Curing Light Guilin Woodpecker Medical Instrument Co., Ltd., China) respectively. Under isolation, a hybrid resin composite (Filtek Z250 XT Nano Hybrid, 3M ESPE, USA) was compacted (height:2 mm; diameter:3.5 mm) using cylindrical Teflon mold and all samples were mounted on blocks of acrylic resin [DPI RR Cold Cure, Dental Products of India (DPI), India] were stored in distilled water at 37 °C for 24 h followed by thermocycling (1,000 cycles, dwell time of 30s from 5 °C to 55 °C) (12).

### Shear bond strength test

Specimen mounted on the testing jig of a Universal Testing Machine (Instron 3366, Norwood, MA, USA) were subjected to a shear load applied to the bonded interface at a cross-head speed of 0.5 mm/min until failure occurred. An independent operator blinded to the group allocation of the specimens recorded the shear bond strength measurements and documented the SEM observation of failure modes of fracture under three categories: adhesive failure, mixed failure, and cohesive failure (13, 14). The resultant maximum force (N) obtained was recorded and the SBS was calculated using the formula: SBS (MPa)= Maximum Force (N)/Bonded Area (mm2) (15).

### FTIR spectroscopy

IP6 liquid and GSE powder were subjected to FTIR spectrometry (Perkin Elmer spectrum RXI) measurement of the transmittance spectrum in the region between 4,000–500cm^−1^ for characterisation to identify functional groups and confirm the presence of chemical moieties relevant to dentin interaction. Measurements were obtained from the central area of the specimens, ensuring complete coverage of the crystal surface. FTIR spectroscopy was employed to characterize molecular interactions and identify functional groups by analysing vibrational modes, providing insight into the interaction between cross-linking agents and the collagen matrix.

### Statistical analysis

The data was statistically analysed using using IBM SPSS version 21.0. The Shapiro–Wilk test was initially performed to assess the normality of data distribution. As the data demonstrated a normal distribution, one-way analysis of variance (ANOVA) was used to compare mean values among the study groups. Following a statistically significant ANOVA result, Tukey's Honestly Significant Difference (HSD) *post hoc* test was performed for pairwise comparisons to identify differences between individual groups with *p*-value < 0.05 considered statistically significant.

## Results

**Figure 1:**
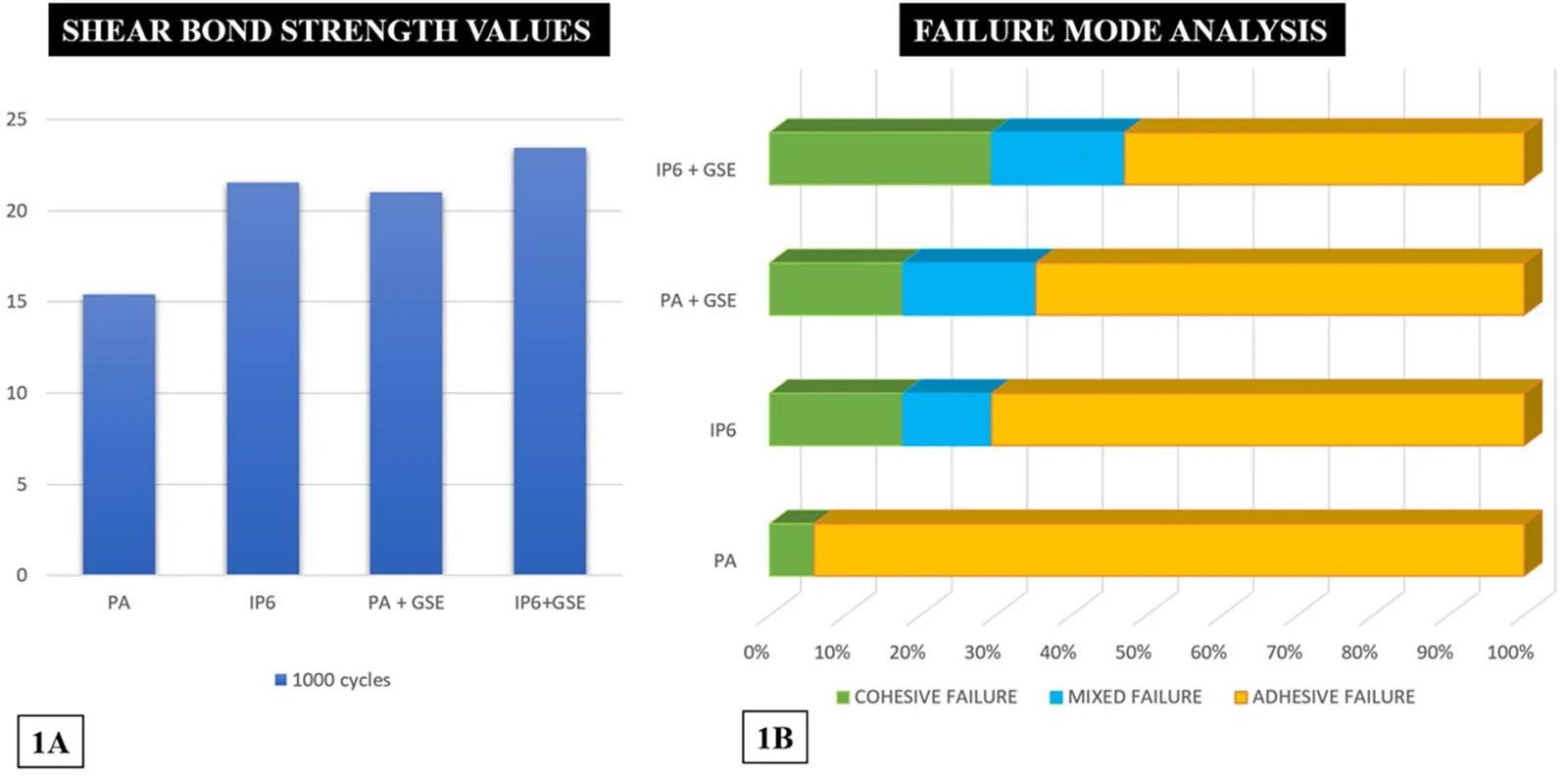
Graph depicting shear bond strength (**1A**) and failure mode analysis (**1B**) of all groups.

Among the test groups, the highest SBS value of 23.44 ± 4.28 MPa was recorded in G4-IP6 + GSE (Figure 1A) followed by 21.51 ± 6.00 MPa (G2-IP6) and 21.01 ± 4.35 MPa (G3-PA + GSE), with the lowest value of 15.40 ± 4.60 MPa recorded in G1-PA, which was the only group that showed a statistically significant difference from the other three groups (*p* < 0.05) (Table 1).

**Table 1:** Mean, standard deviation and significance values in shear bond strength in each tested group *statistically significant difference of *p* < 0.05 noted.

(I) Group	SBS Values (mea*n* ± standard deviation)	(J) Group	Mean Difference (I-J)	Std. Error	95% Confidence Interval
G1-PA	15.40 ± 4.60	G2-IP6	−6.11588*	1.66	−10.5142, −1.7175
		G3-PA + GSE	−5.61118*	1.66	−10.0095, −1.2128
		G4-IP6 + GSE	−8.04176*	1.66	−12.4401, −3.6434
G2-IP6	21.51 ± 6.00	G3-PA + GSE	.50471	1.66	−3.8937, 4.9031
G3-PA + GSE	21.01 ± 4.35	G4-IP6 + GSE	−2.43059	1.66	−6.8289, 1.9678
G4-IP6 + GSE	23.44 ± 4.28	G2-IP6	1.92588	1.66	−2.4725, 6.3242

**Figure 2:**
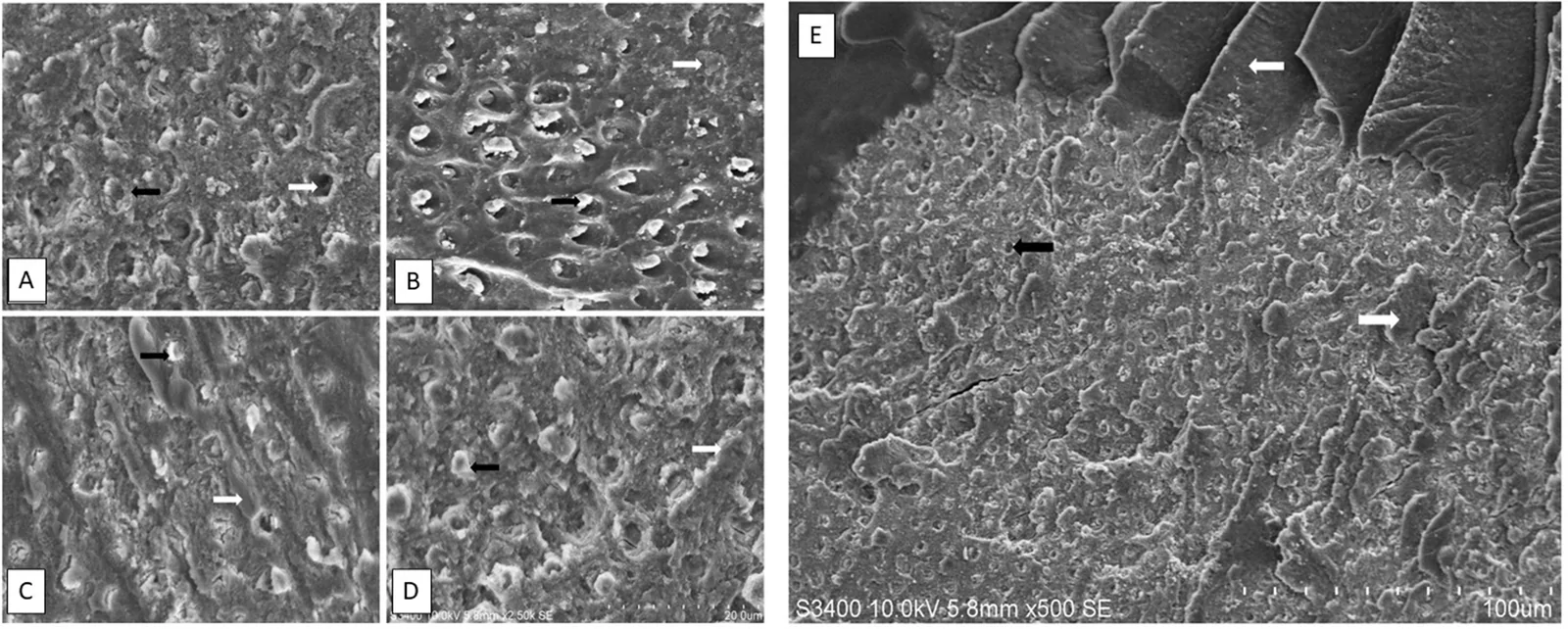
Representative SEM images of the cross-section of the de-bonded interfaces treated with **A**) phosphoric acid with debonding pattern present at the bottom hybrid layer white arrow: dentin tubules, black arrow: peritubular dentin **B**) phytic acid, **C**) phosphoric acid + grape seed extract and **D**) phytic acid + grape seed extract with debonding pattern at the top hybrid layer black arrows: resin tag, white arrows: adhesive resin E) lower-magnification micrograph of phytic acid + grape seed extract with debonding pattern present at the top hybrid layer black arrow: dentin tubule, white arrow: adhesive resin.

In the failure mode SEM analysis, 94.11% of adhesive failures were recorded in G1-PA group followed by 70.58% in G2-IP6, 64.70% in G3-PA + GSE and the lowest of 52.94% were noted in G4-IP6 + GSE (Figure 1B). SEM evaluation further showed distinct differences in the debonding pattern of the hybrid layer. In the G1-PA, only debonding at the bottom was observed with clear exposure of dentinal tubules and peritubular dentin. In contrast, other groups (G2-IP6, G3-PA + GSE, and G4-IP6 + GSE) exhibited debonding primarily at the top of the hybrid layer, with visible resin tags and adhesive remnants along the interface (Figure 2). Notably, G4-IP6 + GSE demonstrated a greater presence of residual adhesive material at the debonded interface. The distribution of failure modes was evaluated descriptively and was not subjected to statistical analysis.

**Figure 3:**
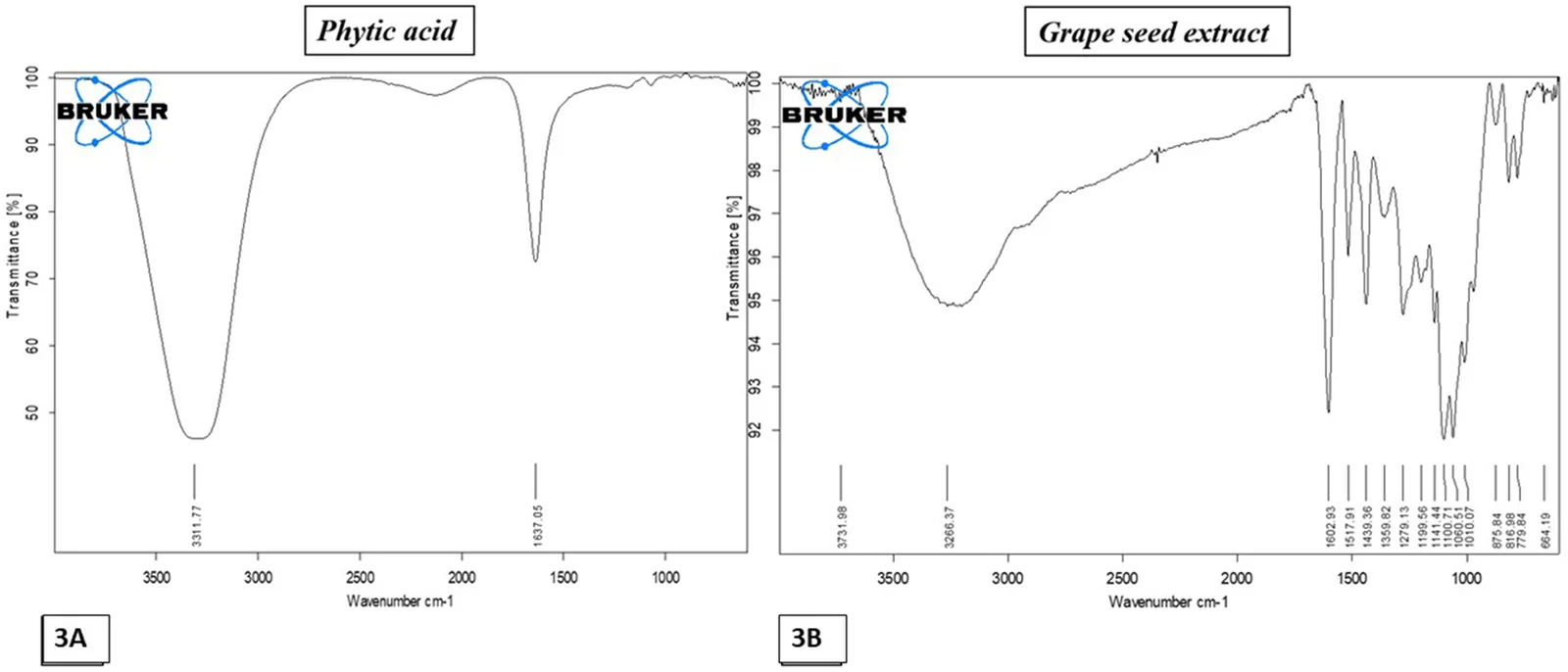
FTIR spectrograph of phytic acid (**3A**) and grape seed extract (**3B**).

The FTIR spectroscopy analysis revealed a broad peak at 3311.77 cm⁻^1^ for IP6 indicating the presence of hydroxyl (-OH) functional group, while GSE displayed a broad peak each at 3266.37 cm⁻^1^ and 1602.93 cm⁻^1^ indicating a hydroxyl and carbonyl group respectively (Figures 3A,B).

## Discussion

Dentin bonding relies on a stable, homogeneous hybrid layer formed by resin infiltration into the demineralized dentin collagen substrate at an average depth of 3–5 μm (16). Depending on the adhesive strategy employed, this depth varies from 1 to 8 μm with the etch-and-rinse systems and 0.5–1 μm with the self-etch systems that produce SBS values typically ranging between 17 and 20 MPa (11, 17, 18).

In the present study, G4-IP6 + GSE demonstrated the highest SBS, followed closely by G2-IP6, G3-PA + GSE, with the lowest SBS noted in G1-PA. A statistically significant difference (*p* < 0.05) was observed between G1-PA and all other groups, indicating a significant effect of these biomodifying agents on dentin bonding, thereby supporting rejection of the null hypothesis.

The control group (G1-PA) exhibited the highest frequency of adhesive failures along with the lowest SBS values, which is in agreement with findings reported by Al-Ammar et al. (11) PA is known to cause aggressive etching of dentin, degrading the collagen underlying the hybrid layer, rendering them susceptible to MMP activation, consequently, deteriorating the resin-dentin bond (19). Apparently, this was further amplified with aging of the specimens as thermocycling tends to speed up the hydrolysis of unprotected collagen fibres, further degrading the resin-dentin bond (7). The lower SBS values could also be attributable to factors like higher thermal expansion rate of restorative materials and repeated contraction/expansion induced gap at the tooth-restoration interface predisposing to pathological fluid movement, microleakage at the bonded area leading to highest adhesive failures post thermocycling (7).

The SBS of G2-IP6 though comparable to the other groups showed a statistically significant difference (*p* < 0.05) with G1-PA alone and a 70% incidence in adhesive failures showed marginally improved SBS suggesting a stronger adhesive bond compared to PA as reported by Attia et al. (9) Souparnika DP et al. also reported that IP6-treated dentin demonstrated higher microshear bond strength following glass ionomer cement (GIC) placement compared with 10% polyacrylic acid and 10% ethylenediaminetetraacetic acid (EDTA) (20). The strong negative charge of IP6 has the ability to chelate positively charged multivalent cations and effectively condition dentin, thus demonstrating comparable results to the traditional PA (9). Consistent with this statement, Anastasiadis K et al. reported comparable dentin surface roughness values following IP6 and PA treatment of coronal dentin (21). It may also function as a collagen crosslinker by forming a hydrogen bond between the hydroxyl and amine groups of the side chain amino acid in collagen as well as bonding with calcium and proteins creating a ternary complex of protein-Ca-Phytate, increasing stability and decreasing susceptibility to collapse (9). In addition to this, the reduced dentinal host-derived endogenous enzymatic activity associated with IP6 can ultimately reduce collagen degradation, thus, improving the bond strength (22, 23). Consistent with this proposed mechanism, a study reported that IP6 pretreatment resulted in lower microleakage and higher shear bond strength (SBS) of resin-modified glass ionomer cement compared with Er,Cr laser and polyacrylic acid conditioning, further supporting its potential as an effective dentin surface conditioner (24).

The G3-PA + GSE group demonstrated SBS values and adhesive failure patterns comparable to those observed in the G2-IP6 and G4-IP6 + GSE, with no statistically significant differences among them (*p* < 0.05). This suggests that the incorporation of GSE enhanced the bond strength of PA-conditioned dentin, consistent with earlier reports (11). When GSE is applied to PA-treated dentin, it interacts with proteins in the underlying unprotected collagen layer to induce cross-links by four different mechanisms: covalent interaction, ionic interaction, hydrogen bonding interaction, or hydrophobic interactions (11). In addition, it is reported to effectively inhibit endogenous dentin proteases activity such as MMP-2, MMP-9 and cysteine cathepsins, thereby decreasing dentin degradation over time (11, 25). However, this mechanism may be limited by the large molecular size of GSE, which is rich in proanthocyanidins composed mainly of catechin, epicatechin, and epigallocatechin subunits and thus, such bulky oligomeric structures can hinder effective penetration into the collagen matrix, minimizing their full potential (26).

The marginally higher SBS value of G2-IP6 compared to G3-PA + GSE although statistically not significant (*p* < 0.05), could be attributed to the effect of conditioning of dentin combined with stabilizing effect on collagen. In addition, the aggressive demineralizing effect of PA during the conditioning stage on G3-PA + GSE maybe instrumental in lowering the SBS values and an increase in the adhesive failures after thermocycling.

G4-IP6 + GSE showed highest SBS which was not statistically significant (*p* < 0.05) compared to the other groups, reflected in the failure mode analysis of this group, indicating a synergistic collagen crosslinking effect of the two different agents i.e., IP6 and GSE, binding to different functional groups of the fibrils (9).

SEM evaluation of failure modes revealed a higher incidence of adhesive failures in the G1-PA samples, characterized by clear visibility of dentinal tubules and peritubular dentin, indicating debonding in the bottom hybrid layer in contrast to the other groups. The present results corroborate the observations of Al-Ammar et al, indicating a comparatively weaker interface which can be attributed to the aggressive nature of PA on the collagen fibrils resulting in reduced intermingling of the collagen fibrils and the resin tags and may be responsible for recording low SBS values (11).

SEM cross-sectional images of G2-IP6 showed resin tags within dentinal tubules along with adhesive resin on the surface of the interface, indicating debonding at the top hybrid layer, implying a superior bond strength compared with G1-PA. Similar debonding at the top hybrid layer was observed in G3-PA + GSE, which was supported by other studies (11). The SEM examination of G4-IP6 + GSE (Figure 2) shows an abundance of adhesive resin at the de-bonded interface indicating stable adhesion, that could explain the higher cohesive failures in this group.

The FTIR graph of IP6 and GSE exhibited the presence of multiple hydroxyl groups which, based on their chemical characteristics, may facilitate hydrogen-bond interactions with the carbonyl groups of amino acids within type I collagen (16, 27). In IP6, these hydrogen and heteropolar bonds, may potentially mask the enzymatic cleavage sites on collagen, making it harder for collagen-degrading enzymes such as collagenase to break down the tissue (28).

In the present study, shear bond strength was evaluated using a Universal Testing Machine, an effective method for assessing dentin bonding protocols as it concentrates stress at the material interface. The debonded surfaces were subsequently examined under SEM to classify failure modes as adhesive, mixed, or cohesive, providing insight into the weakest region of the bonded interface.

Pertaining to the concentration of IP6, although literature shows both 0.5% and 1% IP6 have been shown to improve resin–dentin bond strength, 1% was selected in this study as it is more widely investigated and has demonstrated consistent effectiveness in smear layer removal and maintenance of bond integrity without excessive demineralization (9). Higher acid concentrations were avoided as they may cause excessive demineralization of intertubular dentin, leading to collagen collapse and a potential reduction in bond strength values (9).

A concentration of 6.5% GSE was selected as it is widely reported as a standard concentration in dental research, demonstrating effectiveness as a natural, biocompatible cross-linking agent for dentin biomodification (29).

An ethanol-based adhesive was used in this study due to its ability to displace water and facilitate the infiltration of hydrophobic monomers into the demineralized dentin, promoting more uniform resin infiltration and potentially enhancing bond durability (30). Filtek nano hybrid composite (Filtek Z250 XT Nano Hybrid, 3M ESPE, USA) was chosen due to its widespread use in similar research, attributed to its superior tensile and flexural strength (11). These enhanced mechanical properties make it a suitable and reliable material for assessing the performance and durability of restorative procedures under simulated clinical conditions.

Through the application of thermal cycles in an *in vitro* setting, the influence of varying coefficients of thermal expansion of restorative materials and their effect on the interface can be examined (31). According to the International Standard Organization (ISO), ISO/TR 11405:1994 guidelines, thermocycling should be performed for a minimum of 500 cycles, hence this study subjected the specimens to 1,000 thermocycling cycles as part of the aging protocol (7).

The results of this study provide preliminary insights into Phytic Acid, a natural compound, for dentin conditioning in restorative dentistry. However, further analysis such as nano leakage assessment and hydroxyproline assay may aid in substantiating the results of this study. Additionally, the evaluation of 2% chlorhexidine for its MMP inhibition properties in long-te rm studies may further enhance understanding of bond durability.

Given that *in vitro* studies cannot replicate true clinical conditions due to the absence of pulpal pressure and dentinal fluid, future studies should aim to incorporate more clinically relevant models along with larger sample sizes to validate and extend these results. Although GSE demonstrated promising results, the 1-hour application period represents a limitation, as the extended exposure time, while allowing prolonged interaction with the dentin collagen matrix, may not be clinically feasible. Future studies should investigate shorter, clinically feasible application times and determine the minimum exposure required for effective collagen stabilization. Furthermore, the dark brown discoloration imparted by GSE may raise aesthetic concerns, particularly in highly visible restorative areas. In addition, the 1,000-cycle thermocycling protocol does not adequately simulate the prolonged aging conditions required to assess long-term resin–dentin interface integrity, hence, future studies using extended aging protocols are warranted to evaluate long-term bond durability and interface stability.

### Clinical significance

The reduced incidence of adhesive failures and more favourable failure patterns observed with IP6- and GSE-treated groups indicate improved integrity of the bonded interface, contributing to enhanced longevity of restorations under intraoral conditions. Additionally, the comparable performance of GSE-treated phosphoric acid groups suggests that collagen cross-linking agents may be beneficial as adjuncts to conventional etching protocols. Clinically, the prolonged interaction afforded by the 1-hour GSE application may have facilitated enhanced collagen stabilization and enzymatic resistance; however, translating this protocol into routine adhesive procedures will require optimization of the application duration.

## Conclusion

Within the limitations of this study, collagen cross-linking agents, phytic acid and grape seed extract, significantly improved the composite–dentin shear bond strength of dentin. Their application appears to enhance the structural integrity of demineralized collagen at the resin–dentin interface, thereby supporting their potential incorporation into adhesive restorative protocols as a promising strategy to enhance dentin bond strength. Notably, phytic acid, when used in combination with grape seed extract, provides dentin conditioning comparable to phosphoric acid, suggesting that phytic acid may serve as a viable alternative to phosphoric acid while providing additional biomodifying potential.

## Data Availability

The raw data supporting the conclusions of this article will be made available by the authors, without undue reservation.
